# Supplementary material for: Genetic Background of Macular Telangiectasia Type 2
Source: Int J Mol Sci. 2025 Jan 15;26(2):684. doi: 10.3390/ijms26020684 (PMC11765629; doi:10.3390/ijms26020684)
Supplement: Supplementary file 1 [file ijms-26-00684-s001.zip › 20241023_Table_S4.pdf]

Table S4. Genetic alternations in patients with MacTel co-occurring with other neurological disorders [12–16].

**Gene symbol**<br>**NCBI ID**	**Gene name**	**Position**	**Reference variant**<br>**Reference**	**HGVS.g**<br>**(GRCh38.p14)**	**HGVS.c**	**HGVS.p**	**Allele frequency**<br>**Major (Total; E-NF)**<br>**Minor (Total; E-NF)**	**Clinical significance**<br>**Variation type and length**<br>**Most severe consequence**
SPTLC1<br>10558	Serine palmitoyltransferase long chain base subunit 1	9q22.31	rs119482081<br>[12,13]	g.92080045C>T	NM_006415.4:<br>c.398G>A	p.Cys133Tyr	C: 1.000*<br>T: 0.000*	Pathogenic<br>SNV, 1 bp<br>Missense variant
			rs119482082<br>[13]	g.92080044A>C	NM_006415.4:<br>c.399T>G	p.Cys133Trp	A: 1.000*<br>C: 0.000*	Pathogenic<br>SNV, 1 bp<br>Missense variant
SPTLC2<br>9517	Serine palmitoyltransferase long chain base subunit 2	14q24.3	rs1594986869<br>[12,14]	g.77555325G>A	NM_004863.4:<br>c.1151C>T	p.Ser384Phe	G: 1.000; 1.000<br>A: <0.001; <0.001	Pathogenic/Likely pathogenic<br>SNV, 1 bp<br>Missense variant
			rs1131691917<br>[15]	g.77576869T>C	NM_004863.4:<br>c.529A>G	p.Asn177Asp	NA	Pathogenic/Uncertain significance<br>SNV, 1 bp<br>Missense variant
			rs864621998<br>[13]	g.77576854C>G	NM_004863.4:<br>c.544G>C	p.Ala182Pro	NA	Pathogenic<br>SNV, 1 bp<br>Missense variant
			NA<br>[13]	g.77576869T>G	NM_004863.3:<br>c.529A>C	p.Asn177His	NA	Likely pathogenic<br>SNV, 1 bp<br>NA
CYP2U1<br>113612	Cytochrome P450 family 2 subfamily U member 1	4q25	rs772400670<br>[16]	g.107947417C>A/<br>G/T	NM_183075.3:<br>c.1168C>A/G/T	p.Arg390Ter	C: 1.000; 1.000<br>T: <0.001; <0.001	Pathogenic<br>SNV, 1 bp<br>Stop gained

Method: whole exome sequencing. The reference is listed beside the variant. *NCBI ALFA allele frequency. Abbreviations: not applicable (NA).
