# Supplementary material for: Genetic Background of Macular Telangiectasia Type 2
Source: Int J Mol Sci. 2025 Jan 15;26(2):684. doi: 10.3390/ijms26020684 (PMC11765629; doi:10.3390/ijms26020684)
Supplement: Supplementary file 1 [file ijms-26-00684-s001.zip › 20241229_Table_S1.pdf]

Table S1. MacTel-associated genetic alternations identified by a candidate-gene screening approach [23, 25, 26].

Gene symbol<br>NCBI ID<br>Reference	Gene name	Position	Reference variant	HGVS.g (GRCh38.p14)	HGVS.c	HGVS.p	Allele frequency<br>Major (Total; E-NF)<br>Minor (Total; E-NF)	Clinical significance<br>Variation type and length<br>Most severe consequence
ATM<br>427<br>[23]	ATM serine/threonine kinase	11q22.3	rs587780612	g.108247086_108247092 AAAGAAA>delGAAA	NM_000051.4: c.1024_1030del	p.Glu343fs	AAAGAAA:<br>1.000; 1.000<br>AAA: <0.001; <0.001	Pathogenic/Likely pathogenic<br>Indel-Deletion, 4 bp<br>Frameshift variant
			rs4986761	g.108254034T>A/C	NM_000051.4: c.2119T>A/C	p.Ser707Pro	T: 0.989; 0.994<br>C: 0.011; 0.006	Benign/Likely benign<br>SNV, 1 bp<br>Missense variant
			rs1800057	g.108272729C>A/G	NM_000051.4: c.3161C>A/G	p.Pro1054Arg	C: 0.978; 0.973<br>G: 0.022; 0.027	Benign/Likely benign<br>SNV, 1 bp<br>Missense variant
			rs1801516	g.108304735G>A/C	NM_000051.4: c.5557G>A/C	p.Asp1853Asn	G: 0.870; 0.856<br>A: 0.130; 0.144	Benign<br>SNV, 1 bp<br>Missense variant
			rs148993589	g.108289727A>C/G/T	NM_000051.4: c.4362A>C/G/T	p.Lys1454Asn	A: 1.000; 0.999<br>C: <0.001; 0.001	Uncertain significance/Benign/Likely benign<br>SNV, 1 bp<br>Missense variant
			rs1800058	g.108289623C>G/T	NM_000051.4: c.4258C>G/T	p.Leu1420Phe	C: 0.984; 0.980<br>T: 0.016; 0.020	Benign/Likely benign<br>SNV, 1 bp<br>Missense variant
			rs2234997	g.108235716T>A	NM_000051.4: c.378T>A	p.Asp126Glu	T: 0.989; 0.999<br>A: 0.011; 0.001	Benign/Likely benign<br>SNV, 1 bp<br>Missense variant
			rs3218695	g.108259051C>A/G/T	NM_000051.4: c.2442C>A/G/T	p.Asp814Glu	C: 0.999; 0.999<br>A: 0.001; 0.001	Benign<br>SNV, 1 bp<br>Missense variant
GSTP1<br>2950<br>[25, 26]	glutathione S-transferase pi 1	11q13.2	rs1695	g.67585218A>G/T	NM_000852.4: c 313A>G/T	p.Ile105Val	A: 0.659; 0.654<br>G: 0.341;0.346	Benign<br>SNV, 1 bp<br>Missense variant

Methods: denaturing high-performance liquid chromatography and direct sequencing, polymerase chain reaction-restriction fragment-length polymorphism [23]; di-deoxy nucleotide sequencing [25, 26]. Abbreviations: European, Non-Finnish (E-NF).
