# Supplementary material for: Genetic Background of Macular Telangiectasia Type 2
Source: Int J Mol Sci. 2025 Jan 15;26(2):684. doi: 10.3390/ijms26020684 (PMC11765629; doi:10.3390/ijms26020684)
Supplement: Supplementary file 1 [file ijms-26-00684-s001.zip › 20241229_Table_S3.pdf]

Table S3. The 22 unique rare variants in *PHDGH* detected in 29 MacTel cases [31].

Variant	HGVS.g (GRCh38.p14)	HGVS.c	HGVS.p	Allele frequency<br>Major (Total; E-NF)<br>Minor (Total; E-NF)	Clinical significance<br>Variation type and length<br>Most severe consequence
NA	g.119721318T>C	c.287T>C	p.Met96Thr	NA	NA<br>SNV, 1 bp<br>Missense variant
rs199809714	g.119723404G>A	NM_006623.4: c.319G>A	p.Ala107Thr	G: 1.000; 1.000<br>A: <0.001; <0.001	Uncertain significance<br>SNV, 1 bp<br>Missense variant
NA	g.119723429T>G	c.344T>G	p.Met115Arg	NA	NA<br>SNV, 1 bp<br>Missense variant
rs1031257536	g.119734649C>G	NM_006623.4: c.526C>G	p.Pro176Ala	C: 1.000; 1.000<br>G: <0.001; <0.001	Uncertain significance<br>SNV, 1 bp<br>Missense variant
rs772598618	g.119734761C>T	NM_006623.4: c.638C>T	p.Thr213Met	C: 1.000; 1.000<br>T: <0.001; <0.001	Uncertain significance<br>SNV, 1 bp<br>Missense variant
rs139063843	g.119735333G>A/C/T	NM_006623.4: c.682G>T	p.Gly228Trp	G: 1.000; 1.000<br>A: <0.001; <0.001	Uncertain significance/Likely benign<br>SNV, 1 bp<br>Missense variant
rs147066269	g.119735357C>T	NM_006623.4: c.706C>T	p.Arg236Cys	C: 1.000; 1.000<br>T: <0.001; <0.001	Uncertain significance<br>SNV, 1 bp<br>Missense variant
rs776544484	g.119735358G>A	NM_006623.4: c.707G>A	p.Arg236His	G: 1.000; 1.000<br>A: <0.001; <0.001	Uncertain significance<br>SNV, 1 bp<br>Missense variant
rs755297912	g.119735381G>A	NM_006623.4: c.730G>A	p.Ala244Thr	G: 1.000; 1.000<br>A: <0.001; <0.001	Uncertain significance<br>SNV, 1 bp<br>Missense variant
rs1432614987	g.119737178C>T	NM_006623.4: c.857C>T	p.Ala286Val	C: 1.000; 1.000<br>T: <0.001; <0.001	NA<br>SNV, 1 bp<br>Missense variant
rs1489498331	g.119737210G>A/T	NM_006623.4: c.889G>A/T	p.Glu297Ter	G: 1.000; 1.000<br>A: <0.001; <0.001	Pathogenic<br>SNV, 1 bp<br>Nonsense variant
rs769256568	g.119740470C>T	NM_006623.4: c.1030C>T	p.Arg344Ter	C: 1.000; 1.000<br>T: <0.001; <0.001	Pathogenic/Likely pathogenic<br>SNV, 1 bo<br>Nonsense variant
rs1652148455	g.119740494G>T	NM_006623.4: c.1054G>T	p.Gly352Trp	G: 1.000*<br>T: 0.000*	NA<br>SNV, 1 bp<br>Missense variant

Continues…

rs772970109	g.119741824T>C	NM_006623.4: c.1136T>C	p.Leu379Pro	T: 1.000; 1.000<br>C: <0.001; <0.001	NA<br>SNV, 1 bp<br>Missense variant
NA	g.119741893T>G	c.1205T>G	p.Leu402Arg	T: 1.000; 1.000<br>G: <0.001; <0.001	NA<br>SNV, 1 bp<br>Missense variant
rs121907988	g.119742870G>A	NM_006623.4: c.1273G>A	p.Val425Met	G: 1.000; 1.000<br>A: <0.001; <0.001	Pathogenic/Uncertain significance<br>SNV, 1 bp<br>Missense variant
rs776122990	g.119743002C>G/T	NM_006623.4: c.1405C>G/T	p.Arg469Trp	C: 1.000; 1.000<br>T: <0.001; <0.001	Uncertain significance<br>SNV, 1 bp<br>Missense variant
rs139764141	g.119743003G>A	NM_006623.4: c.1406G>A	p.Arg469Gln	G: 1.000; 1.000<br>A: <0.001; <0.001	Uncertain significance<br>SNV, 1 bp<br>Missense variant
rs1187169434	g.119743049G>A	NM_006623.4: c.1447+5G>A	NA	G: 1.000; 1.000<br>A: <0.001; <0.001	NA<br>SNV, 1 bp<br>Intron variant
rs121907987	g.119743906G>A/C	NM_006623.4: c.1468G>A/C	p.Val490Met	G: 1.000; 1.000<br>A: <0.001; <0.001	Pathogenic/Likely pathogenic<br>SNV, 1 bo<br>Missense variant
rs587731325	g.119743909C>T	NM_006623.4: c.1471C>T	p.Arg491Trp	C: 1.000; 1.000<br>T: <0.001; <0.001	Uncertain significance<br>SNV, 1 bp<br>Missense variant
NA	g.119743976C>T	c.1538C>T	p.Ser513Phe	C: 1.000; 1.000<br>T: <0.001; <0.001	Uncertain significance<br>SNV, 1 bp<br>Missense variant

Method: whole exome sequencing. *NCBI ALFA allele frequency. Abbreviations: not applicable (NA).
