# Supplementary material for: Genetic Background of Macular Telangiectasia Type 2
Source: Int J Mol Sci. 2025 Jan 15;26(2):684. doi: 10.3390/ijms26020684 (PMC11765629; doi:10.3390/ijms26020684)
Supplement: Supplementary file 1 [file ijms-26-00684-s001.zip › 20250114_Table_S2.pdf]

Table S2. MacTel-associated genomic variants discovered through genome-wide association studies [27, 28, 30].

**Gene symbol**<br>**NCBI ID**	**Gene name**	**Position**	**Reference variant**<br>**Reference**	**HGVS.g**<br>**(GRCh38.p14)**	**HGVS.c**	**HGVS.p**	**Allele frequency**<br>**Major (Total; E-NF)**<br>**Minor (Total; E-NF)**	**Clinical significance**<br>**Variation type and length**<br>**Most severe consequence**
PHGDH<br>26227	Phosphoglycerate dehydrogenase	1p12	**rs146953046**<br>[27]	g.119735449T>C/G	NM_006623.4:<br>c.792+6T>C/G	NA	T: 0.988; 0.986<br>G: 0.012; 0.014	Benign/Likely benign<br>SNV, 1 bp<br>Splice donor region variant
			rs532303<br>[27]	g.119722821A>C/G/T	NA	NA	A: 0.999; 1.000<br>C: <0.001; 0.000	NA<br>SNV, 1 bp<br>Intron variant
			**rs477992**<br>[28]	g.119714953A>C/G/T	NA	NA	A: 0.309; 0.314<br>G: 0.691; 0.686	NA<br>SNV, 1 bp<br>Intron variant
			**rs478093**<br>[28]	g.119712503A>C/G	NA	NA	A: 0.276; 0.309<br>G: 0.724; 0.691	NA<br>SNV, 1 bp<br>5'UTR variant
			rs539708<br>[28]	g.119665880G>A/T	NA	NA	G: 0.691; 0.604<br>T: 0.309; 0.396	NA<br>SNV, 1 bp<br>Intron variant
			rs666930<br>[28]	g.119716347T>A/C/G	NA	NA	T: 0.422; 0.464<br>C: 0.578; 0.536	NA<br>SNV, 1 bp<br>Intron variant
			rs483180<br>[28]	g.119724882C>G	NA	NA	C: 0.730; 0.689<br>G: 0.270; 0.311	NA<br>SNV, 1 bp<br>Splice region variant
PPP2R5A<br>5525	Protein phosphatase 2 regulatory subunit B'alpha	1q32.3	rs2120770†<br>[27]	g.212335663A>C/T	NA	NA	A: 0.806; 0.786<br>C: 0.194; 0.214	NA<br>SNV, 1 bp<br>Intron variant
SLC1A4<br>6509	Solute carrier family 1 member 4	2p14	**rs2160387**<br>[27]	g.64993776T>A/C/G	NA	NA	T: 0.488; 0.573<br>C: 0.512;0.427	NA<br>SNV, 1 bp<br>Intron variant

Continues…

CPS1<br>1373	Carbamoyl-phosphate synthase 1	2q34	**rs1047891**<br>[27]	g.210675783C>A	NM_001875.4:<br>c.4217C>A	p.Thr1406Asn	C: 0.689; 0.687<br>A: 0.311; 0.313	Benign<br>SNV, 1 bp<br>Missense variant
			**rs715**<br>[28]	g.210678331T>A/C	NA	NA	T: 0.711; 0.691<br>C: 0.289; 0.309	NA<br>SNV, 1 bp<br>3'UTR variant
CPS1–ERBB4	Intergenic variant		rs4673553<br>[28]	g.210743655T>A/C/G	NA	NA	T: 0.582; 0.546<br>G: 0.418; 0.454	NA<br>SNV, 1 bp<br>Intergenic variant
GCKR<br>2646	Glucokinase regulator	2p23.2	rs1260326[†]<br>[27]	g.27508073T>C/G	NM_001486.4:<br>c.1337T>C/G	p.Leu446Pro	T: 0.383; 0.402<br>C: 0.617; 0.598	Benign<br>SNV, 1 bp<br>Missense variant
SLC4A7–EOMES	Intergenic variants	3p24.1	**rs9820465**<br>[27]	g.27664807T>A/C	NA	NA	T: 0.706; 0.805<br>C: 0.294; 0.195	NA<br>SNV, 1 bp<br>Intergenic variant
			rs35356316<br>[30]	g.27663663G>A	NA	NA	G: 0.755; 0.814<br>A: 0.245;0.186	NA<br>SNV, 1 bp<br>Intergenic variant
SLC6A20<br>54716	Solute carrier family 6 member 20	3p21.31	**rs17279437**<br>[27]	g.45772602G>A	NM_020208.4:<br>c.596C>T	p.Thr199Met	G: 0.911; 0.897<br>A: 0.089; 0.103	Benign<br>SNV, 1 bp<br>Missense variant
ALDH1L1–KLF15	Intergenic variant	3q21.3	rs9820286*<br>[27]	g.126326052A>G	NA	NA	A: 0.787; 0.892<br>G: 0.213; 0.108	NA<br>SNV, 1 bp<br>Intergenic variant
KLF15<br>28999	KLF transcription factor 15		rs9880406*<br>[28]	g.126330462G>A/C	NA	NA	G: 0.786; 0.808<br>A: 0.214; 0.192	NA<br>SNV<br>Regulatory region variant

Continues…

ENSG00000-271904	Novel transcript variant	5q14.3	**rs73171800** [28, 30]	g.88490187T>C	NA	NA	T: 0.846; 0.901<br>C: 0.154; 0.099	**NA**<br>SNV, 1 bp<br>Intron variant
			rs17478824 [28]	g.88489807C>G/T	NA	NA	C: 0.918; 0.904<br>T: 0.082; 0.096	NA<br>SNV, 1 bp<br>Intron variant
MIR9-2HG 645323	MIR9-2 host gene		**rs17421627** [27, 28]	g.88551768T>G	NA	NA	T:<br>G: 0.050; 0.072	NA<br>SNV, 1 bp<br>Intron variant
			rs73173548 [28]	g.88518153T>G	NA	NA	T: 0.885; 0.911<br>G: 0.115; 0.089	NA<br>SNV, 1 bp<br>Intron variant
TMEM161B–MEF2C	Intergenic variant		rs2194025 [28]	g.88502419G>A/C/T	NA	NA	G: 0.851; 0.901<br>C: 0.149; 0.099	NA<br>SNV, 1 bp<br>Intergenic variant
CD109 135228	CD109 molecule	6q13	rs1973480[†] [27]	g.73802257A>C/G/T	NA	NA	A: 0.669; 0.700<br>G: 0.331; 0.300	NA<br>SNV, 1 bp<br>Intron variant
PSPH 5723	Phosphoserine phosphatase	7p11.2	**rs6955423** [27]	g.56031659G>A/C	NA	NA	G: 0.294; 0.244<br>A: 0.706; 0.756	NA<br>SNV, 1 bp<br>Intron variant
			**rs4948102*** [27]	g.56029572C>A/G/T	NA	NA	C: 0.294; 0.244<br>G: 0.706; 0.756	NA<br>SNV, 1 bp<br>Intron variant
			rs4947534* [28]	g.56011401T>A/C/G	NM_004577.3: c.*361A>G	NA	T: 0. 293; 0.245<br>C: 0.707; 0.755	Benign<br>SNV, 1 bp<br>3'UTR variant
			rs11238389* [28]	g.56012051A>G	NA	NA	A: 0.289; 0.241<br>G: 0.711; 0.759	NA<br>SNV, 1 bp<br>Intron variant
NIPSNAP2 2631	Nipsnap homolog 2		rs4535700* [28]	g.55977755T>C/G	NA	NA	T: 0.286; 0.236<br>C: 0.714; 0.764	NA<br>SNV, 1 bp<br>Intron variant

Continues…

TRIB1AL<br>130406855	TRIB1 associated lncRNA	8q24.21	rs2954021†<br>[27]	g.125469835A>G	NA	NA	A: 0.460; 0.490<br>G: 0.540; 0.510	NA<br>SNV, 1 bp<br>Intron variant
TTC39B<br>158219	Tetratricopeptide repeat domain 39B	9p22.3	**rs677622**<br>[27]	g.15302615A>G/T	NA	NA	A: 0.114; 0.138<br>G: 0.886; 0.862	NA<br>SNV, 1 bp<br>Intron variant
REEP3<br>221035	Receptor accessory protein 3	10q21.3	**rs10995566**<br>[27]	g.63603406C>G/T	NA	NA	C: 0.714; 0.685<br>T: 0.286; 0.315	NA<br>SNV, 1 bp<br>Intron variant
ELP4<br>26610	Elongator acetyltransferase complex subunit 4	11p13	rs2984814†<br>[27]	g.31533417G>A/T	NA	NA	G: 0.698; 0.662<br>T: 0.302; 0.338	NA<br>SNV, 1 bp<br>Intron variant
SNHG16<br>100507246	Small nucleolar RNA host gene 16	17q25.1	rs11077850†<br>[27]	g.76665354T>A/C	NA	NA	T: 0.742; 0.816<br>C: 0.258; 0.184	NA<br>SNV, 1 bp<br>Intron variant
CERS4<br>79603	Ceramide synthase 4		rs36259†<br>[27, 30]	g.8262020G>A/C/T	NA	NA	G: 0.243; 0.239<br>A: 0.757; 0.761	NA<br>SNV, 1 bp<br>Missense variant
FBN3–CERS4	Intergenic variant	19p13.2	**rs139412173**<br>[27]	g.8170367A>G	NA	NA	A: 0.963; 0.949<br>G: 0.037; 0.051	NA<br>SNV, 1 bp<br>Intergenic variant
NFILZ<br>105372267	NFIL3 like basic leucine zipper		rs4804075*<br>[30]	g.8668682G>A/C	NA	NA	G: 0.816; 0.764<br>A: 0.184; 0.236	NA<br>SNV, 1 bp<br>Intron variant

Methods: Illumina Omni SNP chips, TaqMan assays [28, 30]; Illumina Human Omni5-Exome-4 Array, Illumina Global Screening Array, Illumina Omni2.5 BeadChip Array [27]. Bold variants: the most significant variant at the locus. /: no data available. *Variants genotyped with TaqMan assays in the replication stage [28]; †variant at suggestively/conditionally significant locus [27].
